# Risk factors, clinical features, and impact on survival of lung cancer in patients with idiopathic pulmonary fibrosis: A retrospective study

**DOI:** 10.1097/MD.0000000000047714

**Published:** 2026-02-13

**Authors:** Hasibe Çiğdem Erten, Sevda Şener Cömert, Talha Doğruyol, Ali Fidan, Gonca Gül Geçmen, Cihan Akgül Özmen, Sümeyye Kodalak Cengiz, Saibe Fulya Elmastaş Akkuş, Recep Demirhan

**Affiliations:** a Department of Pulmonary Diseases, University of Health Sciences, Kartal Dr. Lütfi Kirdar City Hospital, Istanbul, Turkey; b Department of Thoracic Surgery, University of Health Sciences, Kartal Dr. Lütfi Kirdar City Hospital, Istanbul, Turkey; c Department of Pathology, University of Health Sciences, Kartal Dr. Lütfi Kirdar City Hospital, Istanbul, Turkey; d Department of Radiology, University of Health Sciences, Kartal Dr. Lütfi Kirdar City Hospital, Istanbul, Turkey.

**Keywords:** body mass index, emphysema, idiopathic pulmonary fibrosis, lung neoplasms, smoking

## Abstract

Idiopathic pulmonary fibrosis (IPF) is a chronic progressive lung disease, and lung cancer is a significant comorbidity with high prevalence and adverse impact on survival. Early detection of IPF and targeted interventions require understanding the associated risk factors and clinical presentation of lung cancer in IPF. This single-center, retrospective cohort study aimed to identify risk factors for lung cancer in IPF patients, investigate its clinical features, and determine its impact on survival. Of the 1481 patients with interstitial lung disease, 436 met the criteria for IPF per American Thoracic Society/European Respiratory Society guidelines. Patients followed up for <6 months (n = 31), patients with both IPF and lung cancer (n = 19), and patients whose data were incomplete (n = 18) were excluded from the study. In the end, the study group consisted of 59 patients who developed lung cancer during follow-up, and the control group consisted of 59 randomly selected IPF patients without cancer. Patients’ clinical, radiological, and laboratory data were collected from their medical records. The mean age of the sample, 83.9% of which was male, was 66.9 ± 8.3 years. Emphysema, low body mass index, absence of antifibrotic therapy, presence of weight loss symptoms, and ≥36 pack-years of smoking were significant risk factors for lung cancer (*P* <.05), as also confirmed by multivariate analysis. Squamous cell carcinoma was the most common histological type (45.8%), with lower lobe predominance (59.3%) and peripheral location (78.0%). Most tumors (78.0%) were within or adjacent to fibrotic tissue. The median time from IPF diagnosis to lung cancer development was 2.80 years. The 0 to 3, 3 to 5, and >5-year mortality rates for patients with and without lung cancer were 15.2% to 3.3%, 35.5%- 8.4%, and 42.3% to 15.2%, respectively (*P* = .026). Our study identified significant risk factors for lung cancer in IPF patients and demonstrated its negative impact on survival. The presence of emphysema, low body mass index, absence of antifibrotic therapy, and ≥36 pack-years of smoking were significantly associated with lung cancer development. Awareness of these factors is crucial for early diagnosis and appropriate treatment strategy determination, potentially improving outcomes in this high-risk population.

## 1. Introduction

Idiopathic pulmonary fibrosis (IPF) is a long-term, progressive lung disease characterized by the development of fibrosis with no known cause, usually seen in elderly individuals. It is histologically and radiologically linked to the usual interstitial pneumonia (UIP) pattern. IPF is the most common disease in the category of idiopathic interstitial pneumonia. The incidence of IPF increases with age. Most IPF patients have a history of smoking. The most common symptoms of IPF are exertional dyspnea, cough, bibasilar inspiratory crackles, and clubbing.^[1]^ The incidence of IPF is estimated to be between 0.09 and 1.30 per 10,000 people, depending on the country.^[2]^ Advanced age, smoking history, low body mass index (BMI), pulmonary hypertension, extensive radiological involvement, comorbidities, and exacerbations were reported as the risk factors for poor prognosis of IPF. The survival time of patients with IPF, which has a variable course, ranges between 2.5 and 5 years.^[3]^ Although the poor prognosis of IPF is mainly due to respiratory failure resulting from progressive fibrosis, comorbidities also play a significant role in the progression of the disease.

One of the most significant comorbidities in patients with IPF is lung cancer. The prevalence of lung cancer in patients with IPF ranges between 2.8% and 48.2%.^[4]^ Although the exact mechanism of lung cancer development in IPF patients is not fully understood, advanced age, male gender, smoking history, and the presence of emphysema have been reported as significant risk factors for carcinogenesis.^[5–7]^ Pulmonary fibrosis itself is also considered an independent risk factor for the development of lung cancer.^[8,9]^ The significance of lung cancer in patients with IPF stems from both its high prevalence and its negative impact on survival.^[10,11]^ Furthermore, the coexistence of lung cancer and IPF presents substantial challenges in diagnosis and treatment. Therefore, identifying the risk factors and clinical features of lung cancer in patients with IPF is crucial for early diagnosis and disease management through the development and use of appropriate treatment approaches. Despite its importance, there are limited studies on lung cancer in patients with IPF. Moreover, the existing studies have been conducted with small sample sizes and were limited to specific patient populations.^[12,13]^ In light of this information, the objective of this study is to identify the risk factors for lung cancer in patients with IPF, investigate its clinical features, and determine its impact on survival.

## 2. Material and methods

### 2.1. Study design

This study was designed as a retrospective, single-center, cohort study. The study protocol was approved by the ethics committee of Kartal Dr Lütfi Kirdar City Hospital (Approval Number: 2024/010.99/3/20). Written informed consent was obtained from all patients.

### 2.2. Population and sample

The study population consisted of 1481 patients assigned the diagnostic code for interstitial lung disease at a tertiary care hospital between January 1st, 2017, and December 31st, 2023. Of these patients, 436 met the IPF criteria according to the American Thoracic Society/ European Respiratory Society guidelines.^[14]^ Patients with a follow-up duration of less than 6 months (n = 31), those diagnosed simultaneously with IPF and lung cancer (n = 19), and patients with missing data (n = 18) were excluded from the study. In the end, 59 patients who developed lung cancer during follow-up constituted the study group, and the randomly selected 59 IPF patients without cancer constituted the control group.

### 2.3. Data collection

Patients’ data, including demographic characteristics, comorbidities, respiratory symptoms, physical examination findings, spirometry and carbon monoxide diffusion test results, BMI values, 6-minute walk test results, and antifibrotic therapy history within the first 3 months after IPF diagnosis, were obtained from their medical records. In patients diagnosed with lung cancer, the size, location, morphology, radiological density, and relationship with fibrosis of the malignant lesion were assessed and recorded. Survival data were obtained from patients’ medical records and through telephone interviews with the patients.

### 2.4. IPF diagnosis

All patients were diagnosed with IPF according to the American Thoracic Society/ERS guidelines. To this end, images of high-resolution computed tomography (HRCT) scan of the chest performed at the time of diagnosis were evaluated by a thoracic radiologist for each patient. The radiological findings were categorized as UIP, possible UIP, or indeterminate UIP.

### 2.5. Emphysema diagnosis

The presence of low-attenuation areas separated from normal lung parenchyma by a very thin wall (<1 mm) or without wall formation was considered to indicate emphysema diagnosis.

### 2.6. Lung cancer diagnosis

Lung cancer diagnosis was confirmed pathologically in all patients based on biopsy and/or surgical material and classified histopathologically. In all patients who underwent surgery, the UIP pattern was confirmed by a pathologist.

### 2.7. Statistical analysis

Statistical analyses of the collected data were conducted using SPSS Statistics 17.0 (Statistical Package for the Social Sciences for Windows, Version 17.0, SPSS Inc., Chicago, 2008) software package. The results of the statistical analyses were expressed using descriptive statistics, i.e., mean ± standard deviation values in the case of continuous variables and numbers and percentage values in the case of categorical variables. The normal distribution characteristics of numerical variables were analyzed using the Kolmogorov-Smirnov test. The continuous variables were compared using the Mann–Whitney U test, and the categorical variables were compared using Pearson chi-square and Fisher exact tests. Independent variables that significantly predicted lung cancer in patients with IPF were identified using univariate and multivariate analyses. Survival outcomes were assessed using Kaplan–Meier curves and the log-rank test. Probability (*P*) statistics of <.05 were deemed to indicate statistical significance.

## 3. Results

The mean age of the sample, of which 83.9% were male and 16.1% were female, was 66.9 ± 8.3 years. 86.4% of the patients had a smoking history. The most common (46.6%) comorbidity was hypertension, followed by chronic obstructive pulmonary disease (36.4%) and coronary artery disease (32.2%). The clinical characteristics of patients with and without lung cancer are outlined in Table 1. Univariate analysis revealed the absence of antifibrotic therapy, low BMI, and presence of emphysema as significant risk factors for lung cancer in patients with IPF (*P* = .032, *P* = .006, and *P* = .024, respectively). Additionally, when the mean pack-years of smoking of the sample, that is, 36 pack-years, was taken as the cutoff value, it was determined that lung cancer was significantly more common in those with a history of ≥ 36 pack-years of smoking (*P* = .004). A significant relationship was also found between weight loss symptoms and the development of lung cancer (*P* = .025). Among other risk factors examined, heart failure and the presence of an indeterminate UIP pattern on HRCT were also found to almost significantly predict the development of lung cancer (*P* = .052 for both cases). Further analysis of the factors found to significantly predict lung cancer development in patients with IPF in univariate analysis with multivariate analysis confirmed the significance of these independent variables, i.e., absence of antifibrotic therapy, low BMI, presence of emphysema, ≥36 pack-years of smoking history, and weight loss symptoms as significant risk factors for lung cancer in patients with IPF (*P* = .046, *P* = .004, *P* = .016, *P* = .006, and *P* = .037, respectively) (Table 2).

**Table 1:** Comparison of clinical characteristics, comorbidities, and outcomes between patients with and without lung cancer.

Characteristics	Patients with lung cancer (n = 59)	Patients without lung cancer (n = 59)	*P*-value
Age	67.9 ± 9.1	65.9 ± 7.5	.171
Sex (Male)	49 (83.1%)	50 (84.7%)	.873
Smoking status			
Ex-smoker	42 (71.2%)	35 (59.3%)	.144
Active smoker	10 (16.9%)	15 (25.4%)	
Nonsmoker	7 (11.9%)	9 (15.2%)	
Comorbidities			
Hypertension	27 (45.8%)	28 (47.4%)	.884
Diabetes	14 (23.7%)	9 (15.2%)	.184
COPD	22 (37.3%)	21 (35.5%)	.394
Coronary artery disease	18 (30.5%)	20 (33.8%)	.519
Heart failure	4 (6.8%)	0 (0%)	.052
Symptoms			
Dyspnea	43 (72.9%)	43 (72.8%)	.359
Cough	43 (72.9%)	40 (67.7%)	.180
Weight loss	17 (28.8%)	1 (1.69%)	**.025**
Physical examination findings			
Clubbing	12 (20.3%)	15 (25.4%)	.405
Velcro crackles	35 (59.3%)	35 (59.3%)	.406
Antifibrotic Therapy	22 (37.3%)	48 (81.3%)	**.032**
Radiological pattern			
UIP and possible UIP	50 (84.7%)	56 (94.9%)	.052
Indeterminate UIP	9 (15.3%)	3 (5.0%)	
Emphysema	40 (67.8%)	30 (50.8%)	**.024**
BMI (mean ± SD)	25.9 ± 4.2	28.2 ± 3.8	**.006**
6-min walk test (m) (mean ± SD)	423.4 ± 77.8	488.0 ± 143.6	.179
Ejection fraction % (Mean ± SD)	59.9 ± 6.1	61.9 ± 4.0	.180
Pulmonary function test results			
FVC % (mean ± SD)	86.9 ± 24.0	90.6 ± 19.0	.172
FEV1 % (mean ± SD)	83.7 ± 19.3	88.4 ± 14.5	.114
FEV1/ FVC (mean ± SD)	75.5 ± 8.5	77.7 ± 7.8	.290
DLCO % (mean ± SD)	57.5 ± 20.1	61.9 ± 15.4	.118
Mortality	25 (42.4%)	9 (14.9%)	**.001**
Histopathological type			
Squamous cell carcinoma	27 (45.8%)	–	–
Adenocarcinoma	18 (30.5%)	–	–
Nonsmall cell lung cancer	7 (11.8%)	–	–
Small cell lung cancer	2 (3.4%)	–	–
Large cell carcinoma	2 (3.4%)	–	–
Adenosquamous carcinoma	1 (1.7%)	–	–
Large and small cell carcinoma	1 (1.7%)	–	–
Pleomorphic carcinoma	1 (1.7%)	–	–

All values are presented as mean ± standard deviation (SD) for continuous variables and as n (%) for categorical variables. Bold *P*-values indicate statistical significance at *P* <.05.

BMI = body mass index, COPD = chronic obstructive pulmonary disease, DLCO = diffusing capacity of the lung for carbon monoxide, EF = ejection fraction, FEV1 = forced expiratory volume in 1 second, FVC = forced vital capacity, UIP = usual interstitial pneumonia.

**Table 2:** Univariate and multivariate cox regression analysis identifying risk factors for lung cancer development in IPF patients.

Parameters	Univariate analysis *P*-value	Multivariate analysis *P*-value
Antifibrotic therapy	**.032**	**.046**
Emphysema	**.024**	**.016**
Low BMI	**.006**	**.004**
≥ 36 pack-yr smoking	**.004**	**.006**
Weight loss	**.025**	**.037**

All *P*-values represent the results of Cox regression analysis. Bold *P*-values indicate statistical significance at *P* <.05.

BMI = body mass index, IPF = idiopathic pulmonary fibrosis.

The mean follow-up duration of the patients was 4.03 ± 1.84 years. During the follow-up period, 16% of the patients were diagnosed with lung cancer. Histopathological examination revealed squamous cell carcinoma in 45.8% (n = 27) of the patients and adenocarcinoma in 30.5% (n = 18). Seven (11.8%) patients with nonsmall cell lung cancer could not be subtyped due to insufficient material. Two patients each were diagnosed with small cell lung cancer and large cell lung cancer. One patient each had Adensquamous carcinoma, a combination of small and large cell carcinoma, and pleomorphic carcinoma (Table 1).

**Figure 1.**
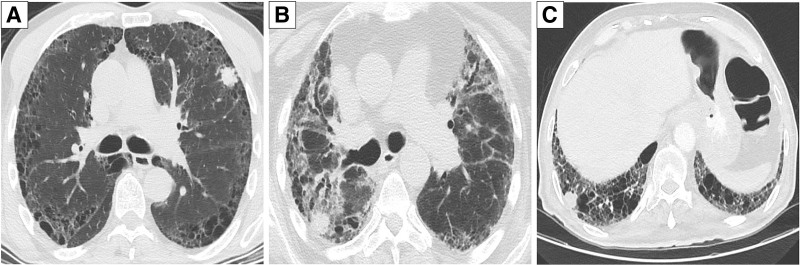
(A–C) Nodules developing on an interstitial background in high-resolution computed tomography.

In terms of the anatomical location of lung cancer, radiological studies revealed that it was located in the lower and upper lobes in 59.3% (n = 35) and 30.5% (n = 18) of the patients, respectively, while it was peripherally in 78.0% (n = 46) of the patients. The morphological appearance of the lesions on HRCT showed that nearly half had irregular borders, and 89.8% were solid in structure. The lesion in78.0% (n = 46) of the patients was within or adjacent to interstitial fibrosis (Fig. 1).

**Figure 2.**
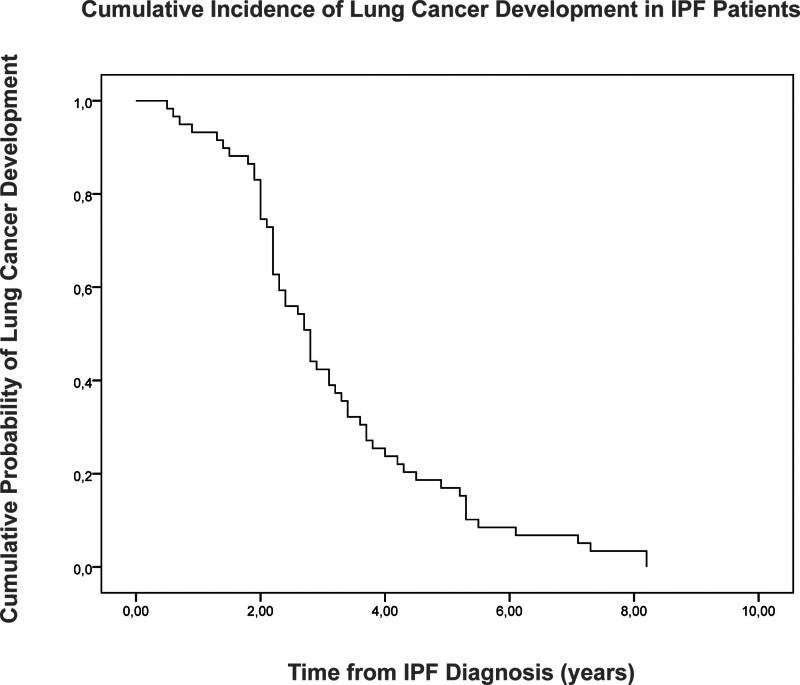
Kaplan–Meier analysis showing the cumulative incidence of lung cancer development in patients with IPF over time. IPF = idiopathic pulmonary fibrosis.

The median time from the diagnosis of IPF to the development of lung cancer was 2.80 ± 0.22 years (95% confidence interval: 2.37–3.23). Of the IPF patients diagnosed with lung cancer, 7.4% were diagnosed by the end of the first year, 18.5% by the end of the second year, 64.8% by the end of the third year, and 92.5% by the end of the fifth year (Fig. 2).

50.8% (n = 30) of IPF patients with lung cancer were in early stage at diagnosis, 18.6% (n = 11) in locally advanced stage and 30.5% (n = 18) in advanced stage. 52.5% (31) of the patients underwent surgery for lung cancer.

**Figure 3.**
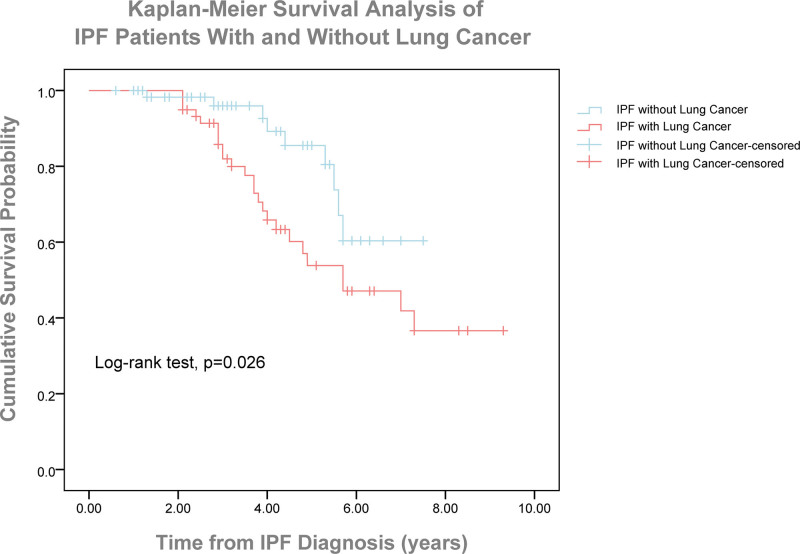
Kaplan–Meier survival curves comparing overall survival between IPF patients with and without lung cancer (log-rank test, P = .026). IPF = idiopathic pulmonary fibrosis.

The median survival of patients with lung cancer was 5.70 ± 1.19 years (95% confidence interval: 5.32–6.9). The 0 to 3, 3 to 5, and >5-year mortality rates for patients with and without lung cancer were 15.2% and 3.3%, 35.5% and 8.4%, 42.3% and 15.2%, respectively. The log-rank test revealed a statistically significant difference between the survival curves of patients with and without lung cancer (*P* = .026) (Fig. 3).

## 4. Discussion

Our study revealed the presence of emphysema, low BMI, absence of antifibrotic therapy, and ≥36 pack-years of smoking as the risk factors significantly affecting the development of lung cancer in patients with IPF. Additionally, lung cancer was significantly more common in patients presenting with weight loss symptoms. Smoking, male gender, advanced age, and presence of emphysema were also reported in the literature as potential risk factors for lung cancer in patients with IPF.^[5–7,15,16]^ The fact that we excluded the patients who were diagnosed simultaneously with IPF and lung cancer, as did Kato et al, constitutes one of the strengths of our study in terms of identifying the risk factors for lung cancer in patients with IPF. Another highlight of our study is that we found low BMI, which has not been investigated as a risk factor in most studies^[4,7,17]^ and was not found to be significant in the studies where it was investigated,^[11,16]^ to be an independent risk factor for the development of lung cancer in patients with IPF.

Contrary to BMI, the presence of emphysema has been investigated in many studies and identified as a risk factor for lung cancer in IPF patients. In one of these studies, which was conducted with 181 patients, 23 of whom had lung cancer, Tomassetti et al identified emphysema as a risk factor for lung cancer.^[15]^ In another study, the relationship between the presence of emphysema and lung cancer development in IPF patients was investigated based on the emphysema score, formulated as the percentage of lung area affected by emphysema on chest CT.^[17]^ Standardization of emphysema by quantifying emphysema in the form of a score may allow for the role of emphysema in lung cancer in the context of IPF to be compared across studies and assessed more objectively. Another study assessed the prevalence of lung cancer in patients with both pulmonary fibrosis and emphysema (CPFE), revealing a higher incidence of lung cancer in CPFE patients compared to those with pulmonary fibrosis alone.^[18]^

A number of studies have been conducted in recent years on the protective effects of antifibrotic therapy against lung cancer.^[19,20]^ These studies have shown that antifibrotic therapy may slow the progression of cancer.^[20]^ In parallel, we found lung cancer to be significantly more common in patients who did not receive antifibrotic therapy. This finding is noteworthy as it supports the potential role of antifibrotic therapy in reducing the risk of lung cancer.

It is well known that smoking can cause both IPF and lung cancer. One study demonstrated that the risk of lung cancer is higher in patients with IPF, and smoking further increases this risk. However, the same study emphasized that there is a significant risk of lung cancer also in nonsmoking IPF patients.^[15]^ Another study conducted with938 IPF patients found a higher incidence of lung cancer in patients with a smoking history at the time of IPF diagnosis.^[17]^ Similarly, Kato et al found a higher rate of lung cancer among patients with a smoking history of ≥35 pack-years.^[16]^ In comparison, we did not find any significant relationship between being a smoker, ex-smoker, or nonsmoker and the development of lung cancer except for in patients with a smoking history of more than 36 pack-years, indicating a higher risk of lung cancer with greater smoking exposure.

We found the incidence of lung cancer in patients with IPF to be 16%, in line with the rate of lung cancer in IPF patients reported between 2.8% and 48.2% in the literature.^[21]^ One study reported 36 lung cancer cases per 1000 IPF patients annually.^[22]^ Another study that retrospectively evaluated 103 patients with IPF reported the cumulative incidence of lung cancer by the end of the 1^st^, 5^th^, and 10^th^ years to be 3.3%, 15.4%, and 54.7%, respectively.^[23]^ These findings show that the risk of lung cancer significantly increases in IPF patients followed up for along term. We found the median time from the diagnosis of IPF to the development of lung cancer to be 2.80 ± 0.21 years. Of the IPF patients diagnosed with lung cancer in our sample, 7.4% were diagnosed by the end of the first year, 18.5% by the end of the second year, 64.8% by the end of the third year, and 92.5% by the end of the fifth year. The significant increase in the rate of IPF patients diagnosed with lung cancer between the second and third years of IPF is noteworthy.

A meta-analysis that compiled the data of IPF patients with lung cancer from 8 countries reported the most common type of lung cancer in these patients to be squamous cell carcinoma, followed by adenocarcinoma.^[21]^ Many other studies have reported similar findings.^[5,7,10,23]^ Similarly, approximately half of our patients with lung cancer had squamous cell carcinoma. A study investigating the HRCT findings of IPF accompanied by lung cancer demonstrated that lung cancer was mostly localized in the lower lobes, within or adjacent to fibrosis, in peripheral, subpleural areas.^[24]^ Similarly, we found the lung cancer to be localized mostly in the lower lobes, peripheral in location, and within or adjacent to fibrosis. The development of cancer in fibrotic tissue supports the “scar-cinoma” hypothesis.^[25]^

Contrary to several previously held studies which did not find any significant difference between IPF patients with and without lung cancer in survival,^[23,26]^ we, as in several more recently held studies,^[10,15]^ have found that the presence of lung cancer significantly reduced survival in patients with IPF. The negative impact of lung cancer on IPF prognosis may be attributed to the fact that the diagnostic and treatment methods associated with lung cancer lead to disease progression, further deteriorating the patients’ overall health and complicating the management of comorbidities. On the other hand, just as lung cancer may worsen the prognosis of IPF, the inflammatory and fibrotic processes in patients with IPF may also accelerate the progression of lung cancer, thereby shortening survival times.^[27]^

Our study had several key limitations. First, its retrospective design might have introduced inherent biases. Secondly, its single-center design might have limited the generalizability of its findings. Thirdly, its relatively small sample size might have reduced its statistical power. Lastly, the lack of follow-up for some patients during the coronavirus 2019 pandemic period and the inability to perform pulmonary function tests and radiological follow-ups in others due to clinical deterioration and exercise limitations prevented us from determining whether radiological progression and loss of pulmonary function were, in fact, risk factors for the development of lung cancer in IPF patients.

Our study had 2 main highlights. First, the risk factors for the development of lung cancer in IPF patients were identified, including the presence of emphysema, low BMI, absence of antifibrotic therapy, and ≥36 pack-years of smoking. Awareness of these factors may contribute to the early diagnosis of lung cancer in IPF patients and help determine the treatment strategies for these patients. Secondly, it was determined that lung cancer caused a significant decrease in the survival of IPF patients.

## Acknowledgments

There were no contributions from any individuals or institutions outside of the author team in the completion of this study. This study received no financial support and there is no conflict of interest. We would like to extend our gratitude to the Co-Founder of Model Statistics and Clinical Trials Center (www.modelistatistik.com), and the editorial team for their meticulous review and linguistic refinement of our manuscript. We confirm that permission has been given to be acknowledged by name.

## Author contributions

**Conceptualization:** Hasibe Çiğdem Erten, Sevda Şener Cömert, Talha Doğruyol, Ali Fidan, Gonca Gül Geçmen, Cihan Akgül Özmen, Sümeyye Kodalak Cengiz, Saibe Fulya Elmastaş Akkuş, Recep Demirhan.

**Data curation:** Hasibe Çiğdem Erten, Sevda Şener Cömert, Talha Doğruyol, Ali Fidan, Gonca Gül Geçmen, Cihan Akgül Özmen, Sümeyye Kodalak Cengiz, Saibe Fulya Elmastaş Akkuş, Recep Demirhan.

**Formal analysis:** Hasibe Çiğdem Erten, Sevda Şener Cömert, Talha Doğruyol, Ali Fidan, Gonca Gül Geçmen, Cihan Akgül Özmen, Sümeyye Kodalak Cengiz, Saibe Fulya Elmastaş Akkuş, Recep Demirhan.

**Investigation:** Hasibe Çiğdem Erten.

**Methodology:** Hasibe Çiğdem Erten.

**Writing – original draft:** Hasibe Çiğdem Erten.

**Writing – review & editing:** Hasibe Çiğdem Erten, Sevda Şener Cömert, Talha Doğruyol, Ali Fidan, Gonca Gül Geçmen, Cihan Akgül Özmen, Sümeyye Kodalak Cengiz, Saibe Fulya Elmastaş Akkuş, Recep Demirhan.
